# Impact of rheumatoid arthritis on complications and hospital outcomes following reverse shoulder arthroplasty: evidence from 389,135 hospitalizations in the national inpatient sample

**DOI:** 10.1007/s00402-026-06370-9

**Published:** 2026-06-14

**Authors:** Assil Mahamid, Miri Elgabsi, Muhammad Khatib, Hamza Murad, Feras Qawasmi, Eitan Lavon, Sana Zahalka, Ali Yassin, Mustafa Yassin

**Affiliations:** 1 Department of Orthopedics, Hasharon Hospital, Rabin Medical Center, Petah Tikva, Israel; 2 Gray Faculty of Medical & Health Sciences, Tel Aviv University, Tel Aviv, Israel; 3 Department of General Surgery, Hillel Yaffe Medical Center, Hadera, Israel; 4 Tel Aviv Sourasky Medical Center, Tel Aviv, Israel; 5 Department of Orthopaedic Surgery, Hillel Yaffe Medical Center, Hadera, Israel

**Keywords:** Reverse shoulder arthroplasty, Rheumatoid arthritis, National inpatient sample, Hospital outcomes, Perioperative complications

## Abstract

**Objective:**

To evaluate the impact of rheumatoid arthritis (RA) on perioperative complications and hospital outcomes following reverse total shoulder arthroplasty (RTSA) using the National Inpatient Sample (2016–2021).

**Methods:**

Adult patients undergoing RTSA were identified using ICD-10 procedure codes. Outcomes included prolonged length of stay (LOS), high hospital charges, inpatient complications, and mortality. Multivariable logistic regression adjusted for demographic, clinical, and hospital characteristics. Survey weights were applied to generate nationally representative estimates.

**Results:**

A total of 389,135 patients underwent RTSA, including 18,140 (4.66%) with RA. In unadjusted analyses, RA patients had higher rates of prolonged LOS (17.8% vs. 14.8%) and acute blood loss anemia (10.7% vs. 8.6%). After multivariable adjustment, RA remained an independent predictor of prolonged LOS (adjusted OR 1.18, 95% CI 1.14–1.23, *p* < 0.001) and acute blood loss anemia (adjusted OR 1.25, 95% CI 1.18–1.33, *p* < 0.001), but not urinary tract infection. In-hospital mortality was rare and could not be modeled due to zero events in the RA group. RA patients also had slightly longer hospitalizations and higher hospital charges.

**Conclusion:**

RA is independently associated with prolonged hospitalization and increased risk of perioperative blood loss anemia following RTSA. These findings highlight the need for targeted perioperative optimization and blood-management strategies in this high-risk population.

**Supplementary Information:**

The online version contains supplementary material available at 10.1007/s00402-026-06370-9.

## Introduction

Reverse total shoulder arthroplasty (RTSA) is increasingly used in patients with rheumatoid arthritis (RA) as surgical indications for shoulder arthroplasty continue to expand [1, 2]. The prevalence of RTSA among patients with RA is not uniformly reported across the literature; however, available evidence suggests a growing utilization of this procedure in this population [3]. Beauperthuy et al. conducted a nationwide analysis and identified 7232 RA patients who underwent RTSA between 2010 and 2023, underscoring the substantial contemporary use of this procedure [4]. Similarly, Jauregui et al. reported 919 RA patients undergoing RTSA in a large inpatient database study [5]. Collectively, these findings indicate that RTSA has become a common surgical strategy for managing advanced RA-related shoulder pathology.

Postoperative complications following RTSA in patients with RA encompass both medical and implant-related domains and occur at higher rates compared with non-RA populations. Medical complications include increased incidences of urinary tract infections, pulmonary embolism, and transfusion requirements, in addition to higher fall rates and prolonged hospitalizations [4]. Implant-related complications are also more prevalent in RA patients, including prosthesis-related failures, greater tuberosity compromise, and intraoperative fractures. Scapular notching remains a frequent radiographic finding, while postoperative acromial and scapular fractures are reported with notable frequency in this cohort [5–8]. 

Although infection rates are not consistently higher across all studies, periprosthetic joint infection remains a significant concern, occasionally necessitating revision procedures. [9, 10] Less common but clinically relevant complications such as nerve injuries and instability have also been documented [5]. Taken together, these data emphasize the heightened vulnerability of RA patients to a broad spectrum of complications following RTSA, reinforcing the need for tailored perioperative management and vigilant postoperative surveillance.

The objective of this study was to evaluate the association between RA and perioperative complications and hospital outcomes following RTSA using a large, nationally representative database. Primary outcomes were prolonged length of stay and acute blood loss anemia. Secondary outcomes included inpatient complications, hospital charges, and in-hospital mortality. We hypothesized that patients with RA would have higher risks of perioperative complications, longer hospitalization, and increased healthcare resource utilization compared with patients without RA.

## Methods

### Data source

The National Inpatient Sample (NIS), developed by the Agency for Healthcare Research and Quality (AHRQ) as part of the Healthcare Cost and Utilization Project (HCUP), was used as the data source for this study. The NIS is the largest publicly available all-payer inpatient database in the United States and captures approximately 20% of hospital discharges from HCUP-participating hospitals, representing more than seven million unweighted hospitalizations annually. Each record in the NIS represents a single hospitalization (discharge) rather than an individual patient. Discharge-level survey weights provided by HCUP were applied to generate nationally representative estimates in accordance with recommended HCUP methodology. The study period included discharges from 1 January 2016 through 31 December 2021. Missing data were minimal, with the highest proportion observed for race (3.65%). Given the low level of missingness, a complete-case analysis was performed as the primary approach. To ensure robustness, a sensitivity analysis using K-Nearest Neighbors (KNN) imputation (k = 5) was additionally conducted, yielding consistent results across all primary outcomes. Imputation was performed prior to the application of survey weights to preserve the internal structure of the dataset (See Table 1).

**Table 1:** ICD-10 diagnostic and procedure codes used to define study variables

Category	Subcategory	ICD10_codes
Procedures	Replacement of Right Shoulder Joint with Reverse Ball and Socket Synthetic Substitute, Open Approach	0RRJ00Z
	Replacement of Left Shoulder Joint with Reverse Ball and Socket Synthetic Substitute, Open Approach	0RRK00Z
Primary Outcome	Rheumatoid Arthritis	M05.0–9, M06.0–4, M06.8–9, M08.0–4, M08.8–9
Surgical Etiology Comorbidities	Osteoarthritis	M00*, M08*, M10*, M13*, M15*, M16*, M17*, M19*, M86*
	Rotator cuff tear	M66*, M67*, M75*, M79*
	Proximal humerus fracture	M97*, S42*, S52*
	Complication	T81*, T84*, T85*, T86*
	Arthropathies	G56*, M07*, M11*, M12*, M14*
	Avascular necrosis	M87*
	Deformity	M21*, M24*, Q74*, Q77*
	Inflammatory arthritis	M05*, M06*
	Malunion/nonunion	M80*, M81*, M84*, M85*
	Malignancy	C40*, C41A2*, C49*, C79*, C90*, D16*, D48*, Z85*
	Other	A41*, A69*, D57*, D62*, D64*, E03*, E11*, E22*, E27*, E53*, E78*, E86*, E87*, F03*, F17*, F32*, G25*, G40*, G47*, G54*, G89*, G90*, I10*, I11*, I25*, I48*, I50*, I63*, I69*, I95*, I97*, J18*, J44*, J45*, J95*, J96*, K21*, K25*, K56*, L40*, M00*, M18*, M25*, M41*, M54*, M61*, M62*, M65*, M89*, M94*, M96*, N17*, N52*, N99*, R06*, R07*, R09*, R13*, R29*, R73*, S06*, S14*, S43*, S44*, S46*, S49*, S72*, S82*, T40*, T78*, Z01*, Z03*, Z23*, Z42*, Z45*, Z47*, Z68*, Z79*, Z87*, Z89*
	Diabetes	E11-14*
	Hypertension	I10*
	Dyslipidemia	E78 *
	Obstructive Sleep Apnea	G473 *
	Anemia	D64 *
	Alcohol Abuse	F10 *
	Mental Disorders	F *
	Alzheimer’s Disease	G30 *
	Parkinson’s Disease	G20
	Chronic Kidney Disease	N18 *
	Chronic Obstructive Pulmonary Disease	J44 *
	Heart Failure	I50 *

### Cohort definition and selection criteria

The National Inpatient Sample (NIS) database was queried for 2016–2021 to identify adult patients (aged > 18 years) who underwent RTSA. The surgeries were classified based on the presence or absence of RA, using the ICD-10 codes for right (0RRJ00Z) and left (0RRK00Z) reverse total shoulder replacement. In total, 77,827 unweighted RTSA hospitalizations were identified, corresponding to a weighted national estimate of 389,135 RTSA hospitalizations. Because the NIS is a discharge-level database, these estimates represent hospitalizations rather than unique individual patients. The National Inpatient Sample contains fully de-identified, publicly available data. According to the Healthcare Cost and Utilization Project (HCUP) data use agreement, studies using NIS data are not considered human subjects research and therefore do not require Institutional Review Board approval or informed consent.

### Outcome variables (End points)

The primary objective of this study was to assess the impact of RA on outcomes and complications following RTSA. Outcome variables of interest included inpatient mortality, length of stay (LOS), hospital charges, and inpatient postoperative complications. These complications included venous thromboembolism (deep vein thrombosis [DVT] and pulmonary embolism [PE]), surgical site infection, cardiac complications (myocardial infarction, cardiac arrest), respiratory complications (pneumonia, respiratory failure), acute renal failure and urinary tract infection [UTI]. Continuous outcome variables, LOS and hospital charges, were dichotomized. Continuous outcome variables, including length of stay (LOS) and hospital charges, were analyzed both as continuous and categorical variables. For the primary analysis, LOS and charges were dichotomized at the 75th percentile of the cohort distribution to define “prolonged LOS” and “high-end hospital charges,” respectively. This approach is commonly used in large administrative database studies to identify patients with disproportionately high resource utilization and to facilitate clinically interpretable risk modeling. The thresholds used in this study were dataset-specific (LOS > 2 days; charges > $88,404) and derived from the distribution of the study cohort rather than predefined cutoffs. To ensure robustness, continuous analyses using non-parametric comparisons (Mann–Whitney U test) were also performed, yielding consistent findings.

### Exposure variables

The patient-level variables included age, sex, race, primary payer, and comorbidities. Comorbidities of interest comprised hypertension, congestive heart failure, chronic obstructive pulmonary disease, chronic kidney disease, type 2 diabetes mellitus, alcohol abuse, Parkinson’s disease, Alzheimer’s disease, mental disorders, dyslipidemia, chronic anemia, and obstructive sleep apnea (Table 2). Hospital-level variables included hospital bed size, location (urban or rural), teaching status, and geographic region.

**Table 2:** Demographics and hospitalizations characteristics among RA and non-RA patients undergoing reverse shoulder replacement surgery

	Overall (*n* = 389,135)	RA (*n* = 18,140)	non-RA (*n* = 370,995)	*p*-Value
Age (mean (SD))	71.19 (8.46)	70.26 (8.42)	71.24 (8.46)	< 0.0001
Female, n (%)	230,660 (59.28)	13,910 (76.68)	216,750 (58.42)	< 0.0001
Race, n (%)				< 0.0001
White	343,420 (88.25)	15,440 (85.12)	327,980 (88.4)	
Black	20,255 (5.21)	1,220 (6.72)	19,035 (5.13)	
Hispanic	16,110 (4.14)	900 (4.96)	15,210 (4.1)	
Asian or Pacific Islander	2,070 (0.53)	130 (0.72)	1,940 (0.52)	
Native American	1,350 (0.35)	110 (0.6)	1,240 (0.33)	
Other	5,930 (1.52)	340 (1.87)	5,590 (1.5)	
Primary Payer, n (%)				0.0001
Medicare	296,490 (76.19)	14,130 (77.89)	282,360 (76.1)	
Medicaid	10,475 (2.69)	545 (3)	9,930 (2.67)	
Private insurance	63,230 (16.25)	2,835 (15.63)	60,395 (16.28)	
Self-pay	1,710 (0.44)	95 (0.52)	1,615 (0.43)	
No charge	90 (0.02)	10 (0.05)	80 (0.02)	
Other	17,140 (4.4)	5*25* (2.89)	16,615 (4.45)	
Comorbidities, n (%)				
Diabetes	89,035 (22.88)	3,925 (21.64)	85,110 (22.94)	0.0711
Hypertension	227,295 (58.41)	10,715 (59.06)	216,580 (58.38)	0.4200
Dyslipidemia	194,110 (49.88)	8,705 (47.97)	185,405 (49.97)	0.0203
Obstructive Sleep Apnea	70,620 (18.15)	3,610 (19.9)	67,010 (18)	0.0054
Chronic Anemia	20,265 (5.21)	1,345 (7.41)	18,920 (5.09)	< 0.0001
Alcohol Abuse	5,020 (1.29)	185 (1.02)	4,835 (1.3)	0.1610
Mental Disorders	133,560 (34.32)	7,155 (39.44)	126,405 (34.07)	< 0.0001
Alzheimer’s Disease	1,255 (0.32)	60 (0.33)	1195 (0.32)	1.0000
Parkinson’s Disease	4,695 (1.21)	185 (1.02)	902 (0.24)	0.3286
Chronic Kidney Disease	38,355 (9.86)	2,020 (11.13)	36,335 (9.79)	0.0088
COPD	8,507 (2.19)	495 (2.73)	8,012 (2.16)	< 0.0001
CHF	20,315 (5.22)	1,155 (6.36)	19,160 (5.16)	0.0017
Hospital Characteristics				
* Hospital Bed Size*,* n (%)*				0.5430
Small	124,835 (32.08)	5,895 (32.5)	118,940 (32)	
Medium	103,135 (26.5)	4,665 (25.72)	98,470 (26.54)	
Large	161,165 (41.42)	7,580 (41.79)	153,585 (41.4)	
* Hospital Location/Teaching*,* n (%)*				0.4938
Rural	34,425 (8.85)	1,550 (8.54)	32,875 (8.86)	
Urban nonteaching	98,665 (25.35)	4,490 (24.75)	94,175 (25.38)	
Urban teaching	256,045 (65.8)	12,100 (66.7)	243,945 (65.75)	
* Hospital Region*,* n (%)*				0.0095
Northeast	57,615 (14.81)	2,570 (14.17)	55,045 (14.83)	
Midwest	113,170 (29.08)	5,080 (28)	108,090 (29.13)	
South	148,845 (38.25)	7,420 (40.9)	141,425 (38.12)	
West	69,505 (17.86)	3,070 (16.92)	66,435 (17.9)	

### Statistical analysis

All statistical analyses were conducted using Python version 3.10. Statistical significance was defined as a two-tailed p-value ≤ 0.05. AI tools were used solely for language editing and clarity improvement. Baseline demographic, clinical, and hospital characteristics were compared between rheumatoid arthritis (RA) and non-RA hospitalizations using Pearson’s χ² test for categorical variables and the Mann–Whitney U test for continuous variables, given non-normal distributions. Categorical variables were reported as counts and percentages, while continuous variables were summarized as mean ± standard deviation. Multivariable logistic regression models were used to evaluate the association between RA and postoperative outcomes, including prolonged length of stay, acute blood loss anemia, and urinary tract infection. Covariates included demographic, clinical, and hospital-level variables identified a priori and through univariate analysis. RA status was included as the primary independent variable. Survey weights were applied to account for the complex sampling design of the National Inpatient Sample and to generate nationally representative estimates. Model results are reported as odds ratios (ORs) with 95% confidence intervals (CIs). Model performance was assessed using discrimination and calibration metrics. Discrimination was evaluated using the area under the receiver operating characteristic curve (AUC), and calibration was assessed using the Hosmer–Lemeshow goodness-of-fit test. To evaluate robustness, sensitivity analyses using propensity score weighting were performed, yielding consistent results with the primary models. Multicollinearity was assessed using variance inflation factors, with all values below 2.

### Missing data and sensitivity analyses

Missing data were minimal, with the highest proportion observed for race (3.65%). Complete-case analysis was used as the primary approach. To ensure robustness, sensitivity analyses using K-Nearest Neighbors (KNN) imputation (k = 5) were performed. Imputation was conducted prior to the application of survey weights. Results from complete-case and imputed datasets were consistent, with no clinically meaningful differences observed.

## Results

### Demographic and clinical characteristics of RA and Non-RA patients

During the study period, a total of 389,135 RTSA hospitalizations were identified. Of these, 18,140 (4.66%) were diagnosed with RA, while 370,995 (95.34%) were non-RA patients. The mean age of the cohort was 71.19 years (± 8.46), with 59.28% of the patients being female. Table 2 presents the demographic and clinical characteristics of RA and non-RA patients undergoing RTSA.

Our analysis revealed statistically significant associations between RA and non-RA hospitalizations across various comorbidities and complications, as demonstrated by the Chi-Square tests. Significant variables included Prolonged Length of Stay, Gender, Race, Primary Payer, and Surgery Etiology, all with p-values below 0.05. After applying the Bonferroni correction, variables related to Hospital Characteristics were no longer statistically significant.

Cramér’s V indicated that most associations were weak. Prolonged Length of Stay showed a minimal association (Cramér’s V = 0.0178), while Gender and Race exhibited slightly stronger but still weak associations (Cramér’s V = 0.0783 and 0.0227, respectively). Surgery Etiology demonstrated a moderate association (Cramér’s V = 0.0738).

A statistically significant difference in mean age was observed between the two groups (70.26 vs. 71.24 years for RA and non-RA hospitalizations, respectively; *p* < 0.0001), although the absolute difference was small. Female patients were significantly more prevalent in the RA group (76.68% vs. 58.42%; *p* < 0.0001).

A significant difference in racial distribution were noted (*p* < 0.0001). RA hospitalizations had a higher proportion of Black (6.72% vs. 5.13%) and Hispanic (4.96% vs. 4.1%) patients, while White patients were more prevalent in the non-RA group (88.4% vs. 85.12%).

Significant differences in insurance coverage were also observed (*p* < 0.0001). RA hospitalizations were more likely to have Medicare coverage (77.89% vs. 76.1%), while non-RA hospitalizations were more likely to have private insurance (16.28% vs. 15.63%). Regarding comorbidities, RA hospitalizations exhibited higher prevalence of chronic anemia (7.41% vs. 5.09%, *p* < 0.0001), mental disorders (39.44% vs. 34.07%, *p* < 0.0001), chronic kidney disease (11.13% vs. 9.79%, *p* = 0.0088), COPD (2.73% vs. 2.16%, *p* < 0.0001), congestive heart failure (6.36% vs. 5.16%, *p* = 0.0017), and obstructive sleep apnea (19.9% vs. 18.0%, *p* = 0.0054). Other comorbidities showed no significant differences between the two groups.

The hospital characteristics showed no significant differences in hospital bed size (*p* = 0.5430) or hospital location/teaching status (*p* = 0.4938) between RA and non-RA hospitalizations. However, a significant difference was observed in hospital region (*p* = 0.0095), with a higher proportion of RA hospitalizations treated in the South region (40.9% vs. 38.12%).

Surgical indications are summarized in Table 3. Osteoarthritis was the most common indication, accounting for 56.26% of cases overall. The prevalence of this diagnosis was similar between RA hospitalizations (55.6%) and non-RA hospitalizations (56.3%).” Other common indications included rotator cuff tear (17.76% overall) and proximal humerus fracture (9.76%). The differences between the groups were statistically significant (p < 0.001).

**Table 3:** Hospitalization’ primary indication forreverse shoulder replacement surgery by RA group

	Overall (*n* = 389,135)	RA (*n* = 18,140)	non-RA (*n* = 370,995)	*p*-Value
Etiology, *n* (%)				< 0.0001
Osteoarthritis	218,940 (56.26)	10,085 (55.6)	208,855 (56.3)	
Rotator cuff tear	69,100 (17.76)	3,425 (18.88)	65,675 (17.7)	
Proximal humerus fracture	37,980 (9.76)	1,360 (7.49)	36,620 (9.87)	
Complication	24,060 (6.18)	1,200 (6.61)	22,860 (6.16)	
Arthropathies	22,845 (5.87)	1,040 (5.73)	21,805 (5.87)	
Avascular necrosis	2,330 (0.6)	165 (0.9)	2,165 (0.58)	
Deformity	1,675 (0.43)	105 (0.57)	1,570 (0.42)	
Inflammatory arthritis	1,195 (0.31)	375 (2.06)	820 (0.22)	
Malunion/nonunion	1,020 (0.26)	45 (0.24)	975 (0.26)	
Malignancy	335 (0.09)	10 (0.05)	325 (0.08)	

### Univariate analysis of complications and hospital outcomes by RA status

Significant differences were observed between RA and non-RA hospitalizations across several medical complications and hospital outcomes, as shown in Table 4. Prolonged length of stay was more common among RA hospitalizations (17.8% vs. 14.8%; absolute difference 3.0%; OR 1.25, *p* < 0.001). Acute blood loss anemia was more frequent in RA hospitalizations (10.7% vs. 8.6%; absolute difference 2.1%; OR 1.27, *p* < 0.001).

No significant differences were found in respiratory complications, acute renal failure, or embolism between the two groups. RA hospitalizations had similar odds of high-end hospital charges compared to non-RA hospitalizations (OR 0.95, *p* = 0.1651).

**Fig. 1:**
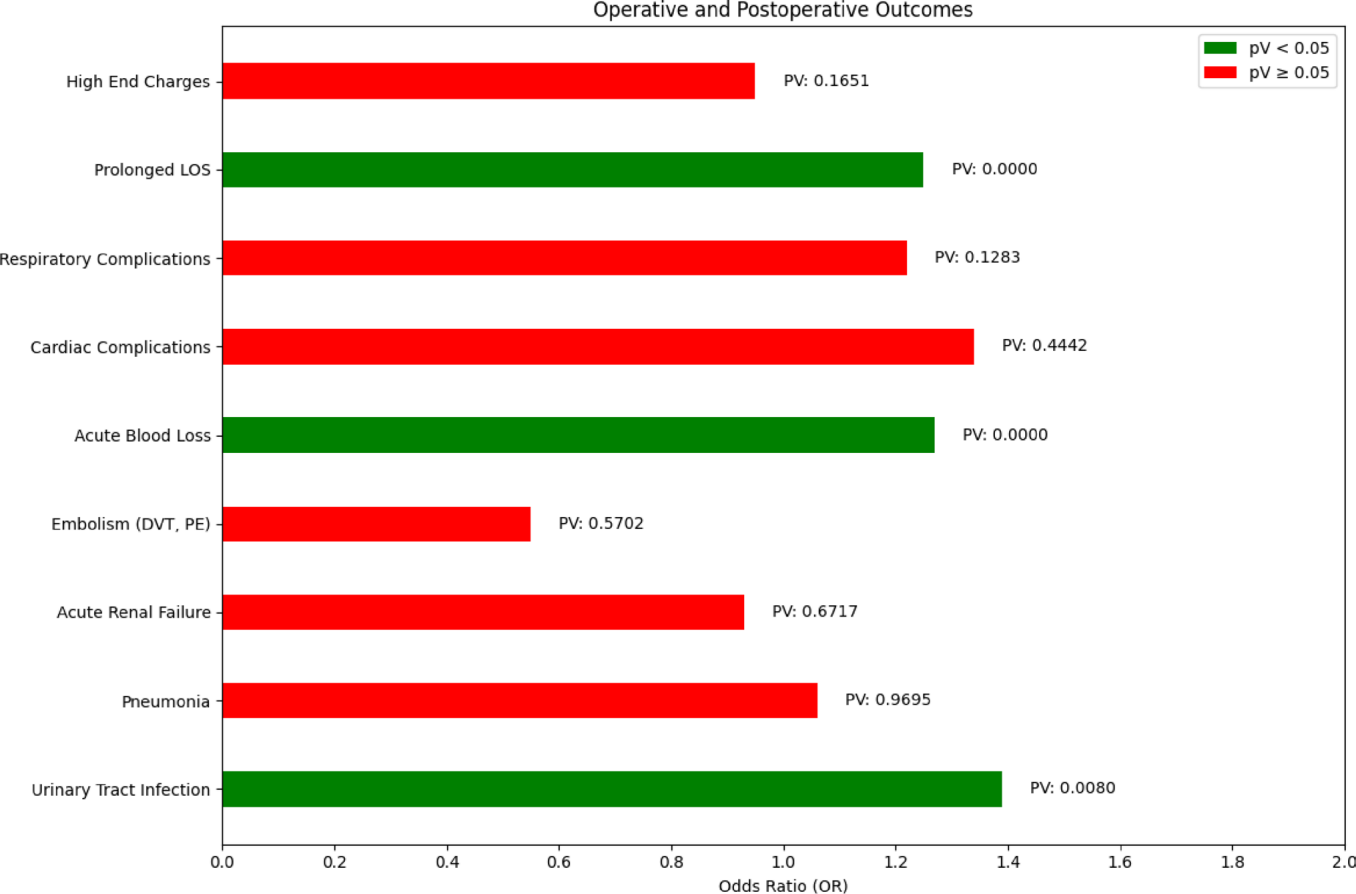
Summarizes the complications and hospitals outcomes comparing RA and non-RA hospitalizations undergoing reverse shoulder replacement surgeries

In-hospital mortality was extremely rare in this cohort. No deaths were observed among RA hospitalizations, whereas 180 deaths (0.04%) occurred in the non-RA group. Given the very low event rate and the relatively small proportion of RA hospitalizations, statistical comparison was not feasible, and the study is underpowered to detect meaningful differences in mortality between groups. Given the low event counts, formal statistical testing (e.g., Fisher’s exact test) would be unlikely to yield reliable or clinically meaningful estimates Fig. 1..

**Table 4:** Summary of medical complications and hospital outcomes comparing RA and non-RA hospitalizations

	RA (*n* = 18,140)	non-RA (*n* = 370,995)	Odds Ratio (95% CI)	*p*-Value
Complications, *n* (%)	2,720 (14.88)	44,815 (12.07)		
Urinary tract infection	375 (2.07)	5,565 (1.5)	1.39 [1.09–1.76]	**0.0080**
Respiratory complications	335 (1.84)	5,620 (1.51)	1.22 [0.95–1.57]	0.1283
Acute renal failure	275 (1.51)	6,015 (1.62)	0.93 [0.71–1.23]	0.6717
Embolism (DVT, PE)	10 (0.05)	370 (0.1)	0.55 [0.14–2.25]	0.5702
Acute blood loss anemia	1,940 (10.69)	31,930 (8.6)	1.27 [1.14–1.42]	**< 0.0001**
Cardiac complications	55 (0.3)	840 (0.22)	1.34 [0.73–2.47]	0.4442
Hospital outcomes				
Prolonged length of stay	3,230 (17.8)	54,850 (14.78)	1.25 [1.14–1.36]	**< 0.0001**
High-end hospital charges	4,325 (23.84)	92,290 (24.87)	0.95 [0.87–1.02]	0.1651
In-hospital mortality	0 (0)	180 (0.04)		

Bold *p*-values indicate statistical significance at α ≤ 0.05

*DVT* deep vein thrombosis, *PE* pulmonary embolism

### Comparison of hospitalization outcomes

**Fig. 2:**
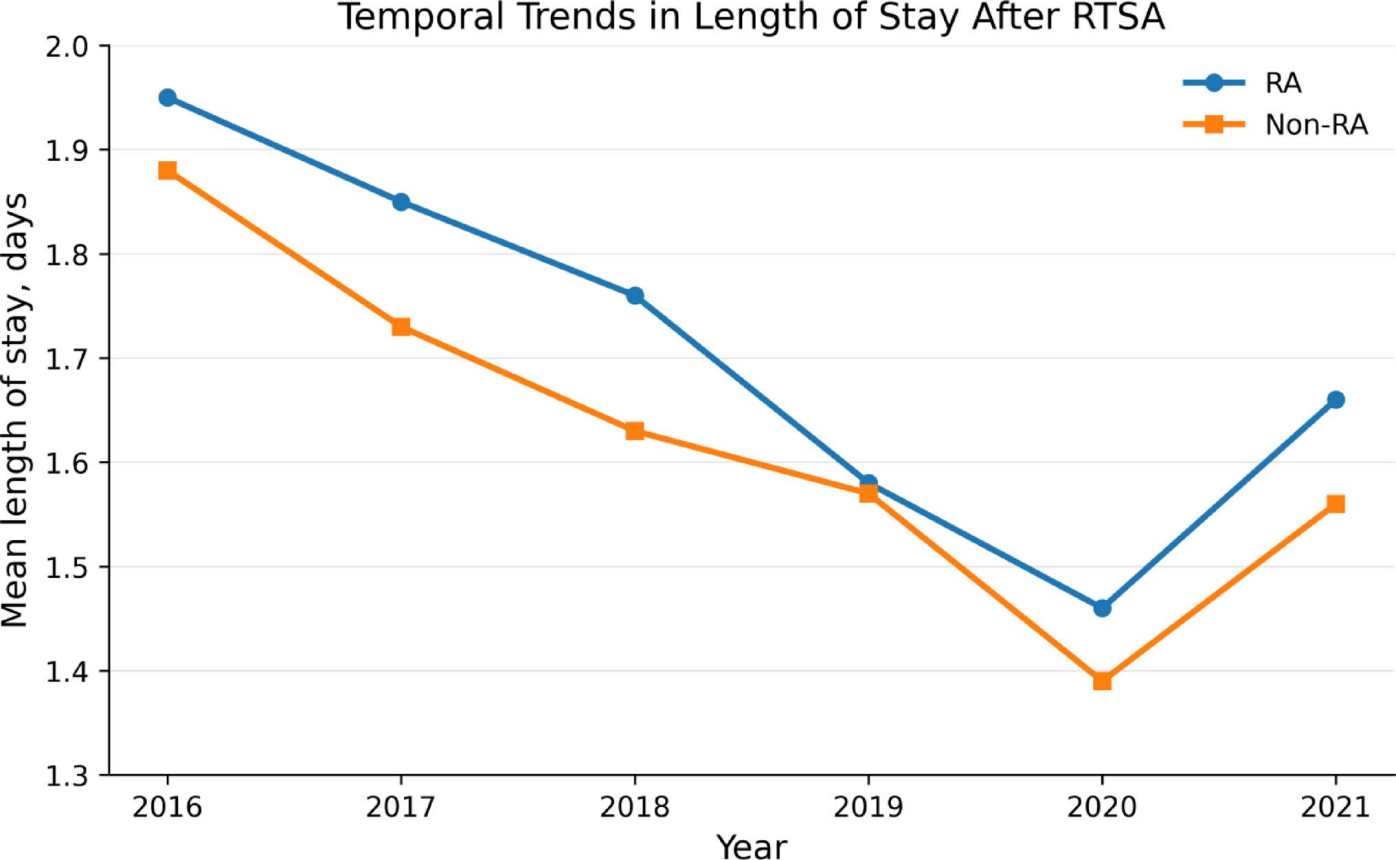
Trends in length of stay over time among RA and non-RA hospitalizations undergoing reverse shoulder arthroplasty

RA hospitalizations incurred slightly higher charges ($78,880) compared to non-RA hospitalizations ($78,115), with a statistically significant difference (*p* < 0.0001). The average length of stay was 1.61 days overall, with RA hospitalizations having a slightly longer stay (1.70 days) compared to non-RA hospitalizations (1.61 days) (*p* < 0.0001), as shown in Table 5 Fig. 2.

**Table 5:** Comparison of hospitalization outcomes between RA and non-RA hospitalizations undergoing reverse shoulder replacement surgeries

	Overall (n = 389,135)	RA (n = 18,140)	non-RA (n =370,995)	*p*-Value
Total charges ($)	78,150 [139-1,5698,76]	78,880 [4,276-1,569,876]	78,115 [139-1,262,381]	<0.0001
Length of stay (days)	1.61 [0-64]	1.70 [0-21]	1.61 [0-64]	<0.0001

RA hospitalizations were associated with slightly higher total charges ($78,880 vs. $78,115; absolute difference $765; *p* < 0.001) and a marginally longer length of stay (1.70 vs. 1.61 days; absolute difference 0.09 days; *p* < 0.001). Although statistically significant, these differences are small in magnitude. These findings were consistent with analyses using continuous outcome measures.

### Multivariable logistic regression results

In the multivariable logistic regression analysis, significant associations were observed between several predictors and the outcomes of LOS, acute blood loss anemia, and UTI. Adjustments were made for key demographic variables (age, gender, race), surgical etiology, primary payer status, and hospital region to control for potential confounding influences. RA was found to be statistically significant for LOS (OR 1.18, *p* < 0.001) and acute blood loss anemia (OR 1.25, *p* < 0.001), but not for UTI (OR 1.21, *p* = 0.0893).

To address the imbalance between the minority and majority groups, particularly for RA, class weighting was applied. After adjusting for class weights, the model more accurately reflected the characteristics of the minority group, providing a more precise estimate of the odds for RA hospitalizations.

**Fig. 3:**
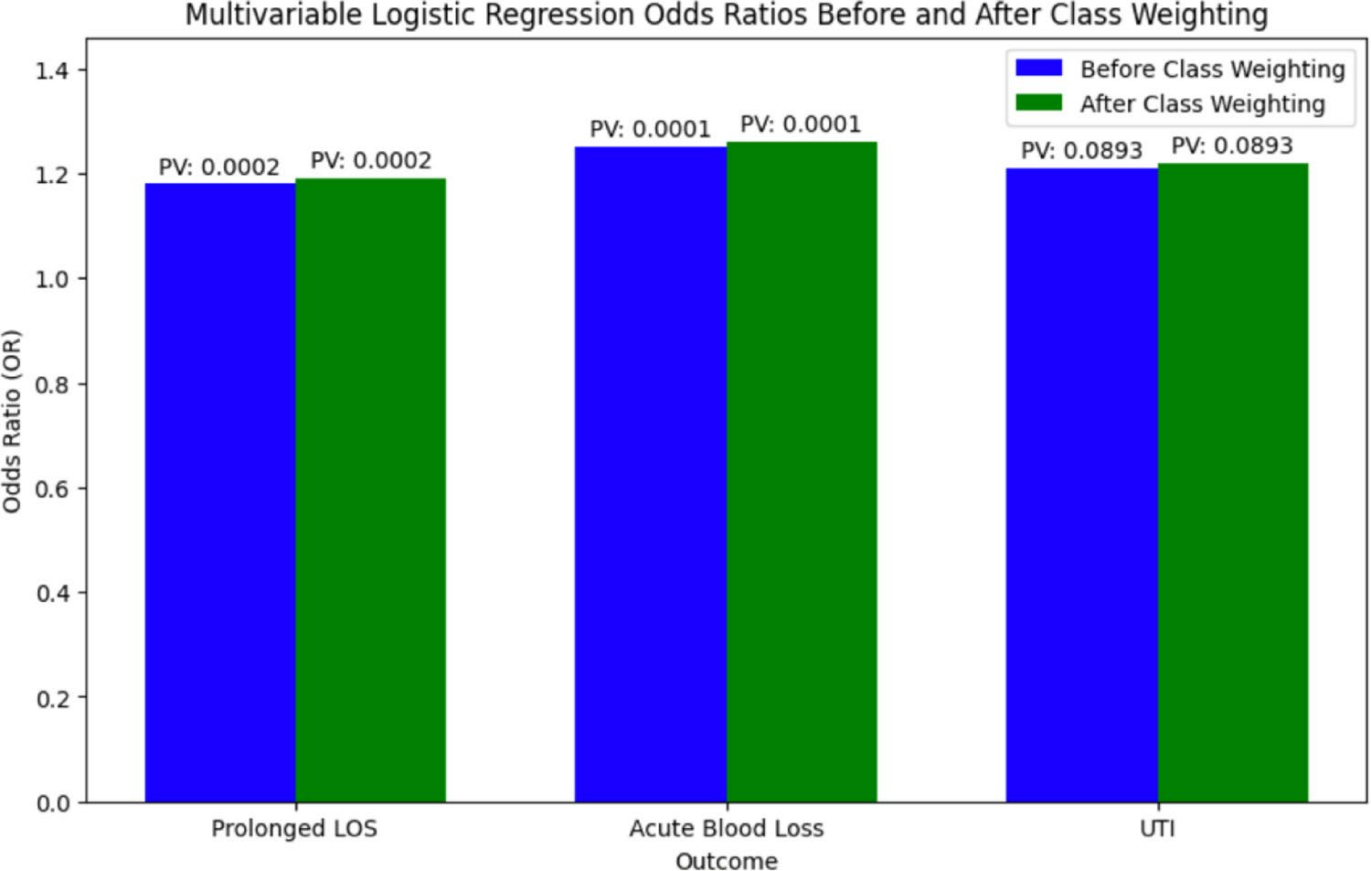
*Multivariable logistic regression odds ratios before and after class weighting for rheumatoid arthritis (RA)* hospitalizations *undergoing reverse shoulder arthroplasty*

For prolonged LOS, RA hospitalizations had an odds ratio (OR) of 1.18 vs. 1.19 (*p* < 0.001) before and after class weighting, respectively. For acute blood loss anemia, the OR for RA hospitalizations was 1.25 vs. 1.26 (*p* < 0.001) before and after class weighting, indicating a slightly stronger association after adjustment. Regarding UTI, RA remained not statistically significant (*p* = 0.0893). Sensitivity analyses using KNN-imputed datasets demonstrated consistent effect estimates and did not materially alter the direction or significance of the associations. The multivariable models demonstrated acceptable discrimination, with AUC values ranging from 0.68 to 0.74 across outcomes. Calibration assessment using the Hosmer–Lemeshow test indicated good model fit (*p* > 0.05 for all models). Sensitivity analyses using propensity score weighting yielded consistent effect estimates, supporting the robustness of the primary findings Fig. 3.

## Discussion

In this large, nationally representative cohort of 389,135 RTSA hospitalizations, RA was present in approximately 5% of cases and was associated with distinct demographic and clinical characteristics. Compared with non-RA hospitalizations, those with RA were more often female, more likely to be Black or Hispanic, and had a higher burden of comorbidities, including chronic anemia, chronic kidney disease, congestive heart failure, chronic obstructive pulmonary disease, and mental health disorders.

RA hospitalizations demonstrated higher odds of prolonged length of stay and acute blood loss anemia; however, the absolute differences were modest (3.0 and 2.1%, respectively), suggesting limited clinical magnitude despite statistical significance. Similarly, RA hospitalizations were associated with slightly longer length of stay and higher hospital charges, although these differences were small and of uncertain clinical relevance. No significant differences were observed in urinary tract infection, respiratory complications, renal failure, or thromboembolism. Importantly, given the large sample size, several statistically significant findings likely reflect small absolute differences and should be interpreted with caution.

These findings are consistent with prior large database studies demonstrating an increased risk of hematologic complications among RA patients undergoing shoulder arthroplasty [4, 11]. This vulnerability is likely multifactorial, reflecting a higher burden of comorbidities, chronic systemic inflammation, and pre-existing anemia, all of which are established predictors of perioperative transfusion and blood loss [11–13]. Additionally, more advanced joint disease and technical complexity may contribute to increased operative demands and perioperative blood loss [12, 13].

Importantly, the observational design of this study precludes causal inference. Although RA was independently associated with selected outcomes, residual confounding remains likely. The NIS database does not capture key disease-specific variables, including RA disease activity, duration, or treatment exposure (e.g., biologic agents or glucocorticoids), which may substantially influence perioperative risk. Therefore, RA may function as a marker of systemic disease burden rather than a direct causal driver of adverse outcomes.

In-hospital mortality was exceedingly rare, with no observed deaths in the RA group and a very low event rate overall. This finding should be interpreted with caution, as the study is underpowered to assess mortality differences, and the absence of events does not imply a protective effect.

A gradual decline in length of stay was observed over time in both groups, consistent with national trends in shoulder arthroplasty driven by improved perioperative protocols, multimodal analgesia, and increased use of outpatient pathways [14–17]. Despite these advances, RA hospitalizations remained associated with slightly longer stays, likely reflecting their greater comorbidity burden and clinical complexity.

Although RA hospitalizations incurred higher costs, the absolute difference was modest and may not represent a clinically meaningful increase in resource utilization at the individual level. This likely reflects a combination of increased perioperative monitoring, transfusion requirements, and comorbidity-related care rather than RA-specific effects alone.

While unadjusted analyses suggested higher rates of urinary tract infection among RA hospitalizations, RA was not an independent predictor after adjustment. This highlights the importance of confounding factors such as comorbidities and demographic characteristics, as well as the role of immunosuppressive therapies, which are not captured in the NIS database but are known to influence infection risk [18–20].

The strengths of this study include the use of a large, nationally representative database and a comprehensive evaluation of inpatient outcomes following RTSA in RA patients, a population that remains underrepresented in the literature.

Several limitations should be considered. As a retrospective analysis of administrative data, the study is subject to residual confounding and cannot establish causality. Complications were identified using ICD-10 codes, which may introduce misclassification, underreporting, and limited ability to distinguish between pre-existing conditions and in-hospital events. Procedure-specific surgical complications are not reliably captured. The NIS includes only inpatient data and does not allow assessment of outpatient procedures or long-term outcomes such as readmissions, revision surgery, or implant survival. Additionally, RA identification is based on coding and lacks information on disease severity, duration, and medication use. The database also does not consistently distinguish between primary and revision RTSA. Finally, percentile-based definitions of prolonged length of stay and high hospital charges are dataset-dependent, although supported by consistent findings in continuous analyses.

In conclusion, RA hospitalizations undergoing RTSA represent a clinically complex subgroup with higher comorbidity burden and increased risk of acute blood loss anemia and prolonged hospitalization. However, the absolute magnitude of these differences is modest. These findings should be interpreted as associative rather than causal and underscore the importance of careful perioperative optimization in this population. Future studies incorporating clinical variables and long-term outcomes are needed to better define risk and guide management.

## Supplementary Information

Below is the link to the electronic supplementary material.

Supplementary Material 1

## Data Availability

No datasets were generated or analysed during the current study.
