# Supplementary material for: Impact of rheumatoid arthritis on complications and hospital outcomes following reverse shoulder arthroplasty: evidence from 389,135 hospitalizations in the national inpatient sample
Source: Arch Orthop Trauma Surg. 2026 Jun 14;146(1):219. doi: 10.1007/s00402-026-06370-9 (PMC13265580; doi:10.1007/s00402-026-06370-9)
Supplement: Supplementary file 1 — Supplementary Material 1 [file 402_2026_6370_MOESM1_ESM.docx]

**Supplement Table:**

**Supplement Table 1:** ICD10 codes of the complications

| **Category** | **Subcategory** | **ICD10_Codes** |
| --- | --- | --- |
| Complication | Surgical Site Infection | T814 * |
|  | Urinary Tract Infection | N39 * |
|  | Pneumonia | J18 *, J15 *, J22 |
|  | Respiratory Failure | J96 * |
|  | Cardiac Arrest | I46 * |
|  | Myocardial Infarction | I20-24 * |
|  | Acute Renal Failure | N17 * |
|  | Embolism (DVT, PE) | I2602, I2609, I2692, I2699, I82401-I82429 |
|  | Acute Blood Loss Anemia (ABLA) | D62 * |
| * Starts with |  |  |
